# Genotype–Phenotype Correlation Model for the Spectrum of *TYR*-Associated Albinism

**DOI:** 10.3390/diagnostics14151583

**Published:** 2024-07-23

**Authors:** Mirjana Bjeloš, Ana Ćurić, Mladen Bušić, Benedict Rak, Biljana Kuzmanović Elabjer

**Affiliations:** 1 University Eye Department, Reference Center of the Ministry of Health of the Republic of Croatia for Inherited Retinal Dystrophies, Reference Center of the Ministry of Health of the Republic of Croatia for Pediatric Ophthalmology and Strabismus, University Hospital “Sveti Duh”, 10000 Zagreb, Croatia; dr.mbjelos@gmail.com (M.B.); mbusic@kbsd.hr (M.B.); benedict.rak@gmail.com (B.R.); belabjer@kbsd.hr (B.K.E.); 2 Faculty of Medicine, Josip Juraj Strossmayer University of Osijek, 31000 Osijek, Croatia; 3 Faculty of Dental Medicine and Health Osijek, Josip Juraj Strossmayer University of Osijek, 31000 Osijek, Croatia

**Keywords:** albinism, pigmentation, depth perception, genotype, phenotype, optical coherence tomography

## Abstract

We present two children aged 3 and 5 years who share identical *TYR* genotype, yet exhibit contrasting phenotypic manifestations in terms of eye, skin, and hair coloration. The patients are heterozygous for *TYR* c.1A>G, p. (Met1?), which is pathogenic, and homozygous for *TYR* c.1205G>A, p. (Arg402Gln), which is classified as a risk factor. The children manifested diminished visual acuity, nystagmus, and foveal hypoplasia. The first patient presented with hypopigmentation of the skin, hair, and ocular tissues, while the second patient presented with hypopigmentation of the skin, hair, retinal pigment epithelium, and choroid with dark brown irises. Furthermore, the brown-eyed subject presented astigmatic refractive error and both global and local stereopsis capabilities, contrasting with the presentation of hypermetropia, strabismus, and the absence of stereopsis in the blue-eyed individual. Herein, we propose a genotype–phenotype correlation model to elucidate the diverse clinical presentations stemming from biallelic and triallelic pathogenic variants in *TYR*, establishing a link between the residual tyrosinase activity and resultant phenotypes. According to our proposed model, the severity of *TYR* variants correlates with distinct albino phenotypes. Our findings propose the potential association between reduced pigmentation levels in ocular tissues and binocular functions, suggesting pigmentation as a possible independent variable influencing the onset of strabismus—an association unreported until now in the existing literature.

## 1. Introduction

Albinism encompasses a spectrum of rare disorders characterized by both clinical and genetic variability, leading to deficiencies in melanin pigment production [1]. This deficiency can arise from disruptions in various stages of melanin synthesis, melanosome maturation, or melanin distribution within melanocytes [2]. Melanin, thought to reduce oxidative stress, initiates its synthesis through the conversion of tyrosine to L-DOPA as catalyzed by tyrosinase [2]. Subsequently, divergent biosynthetic pathways produce eumelanin via enzymes such as tyrosinase-related protein 1 and dopachrome tautomerase, while pheomelanin synthesis is cysteine-dependent [2]. Melanin is then deposited within melanosomes, specialized organelles whose composition and structural variability influence pigmentation in various tissues including ocular, hair, and skin. Melanocytes, originating from neural crest cells, are distributed in these tissues as well as extracutaneous sites like ocular and cochlear tissues [3]. However, melanocytes situated within the stroma and anterior layers of the eye retain melanin within their cytoplasms, whereas, in other regions of the body, melanin is excreted from the cells [4]. This phenomenon elucidates the mechanism behind the development of eye color in infants, contrasting with the dynamic nature of skin pigmentation alterations observed throughout one’s lifetime. Notably, melanin production in the retinal pigment epithelium (RPE) is among the earliest observed during embryonic development due to the early expression of melanogenic genes [5]. In the context of ocular function, melanin metabolism plays a critical role in the histogenesis of retinal pigment, the metabolism of retinal ganglion cells, and the organization of retinal-fugal fibers [6]. Dysfunctional melanin biosynthesis can disrupt embryonic processes, leading to anomalies such as nystagmus and reduced visual acuity at birth by impeding retinal differentiation and optic chiasm decussation [2]. Bakker et al. proposed a human-specific retinal pigmentation pathway for albinism, highlighting the complexity and uniqueness of melanin-related processes in human development and physiology [2].

Oculocutaneous albinism (OCA) is characterized by the reduction or absence of melanin in the skin, hair, and eyes [7]. The non-syndromic OCA is inherited in an autosomal recessive manner and is mainly due to disease-causing variants in one of the following four genes: *TYR* (OCA1), *OCA2* (OCA2), *TYRP1* (OCA3), and *SLC45A2* (OCA4) [8]. The prevalence of all forms of albinism varies worldwide and has been estimated at approximately 1/17,000 [7]. Eye manifestations of OCA include reduced visual acuity, refractive errors, color vision impairment, iris hypopigmentation and translucency, reduced pigmentation of the RPE, foveal hypoplasia (FH), prominent photophobia, and congenital nystagmus [7]. Misrouting of the optic nerves is a characteristic finding, resulting in strabismus and reduced stereoscopic vision [7].

The presentation of OCA demonstrates a subtle interplay with pigmentation thresholds within affected individuals. Those of Black ethnicity, characterized by elevated baseline pigmentation levels, typically require two severe mutations to disrupt the melanin pathways and exhibit observable phenotypic traits [9]. Conversely, individuals with lighter skin tones, such as Caucasians and Hispanics, may showcase a broader spectrum of OCA phenotypes, influenced by ethnic-specific mutations or alleles [9]. OCA subtypes, delineated by melanin biosynthesis, encompass the severe OCA1A phenotype characterized by absent tyrosinase activity and enduring melanin deficiency, as well as milder forms (OCA1B, OCA2, OCA3, and OCA4) where gradual pigment accumulation may alleviate symptoms over time [10]. In contrast, ocular albinism (OA), an X-linked disorder, predominantly affects ocular structures, with mutations in *GPR143*, a gene pivotal in macromelanosome maturation, representing the sole known etiology [11]. The diagnosis of OCA is based on clinical findings of hypopigmentation of the skin and hair, in addition to the characteristic ophthalmological features [1]. Given the clinical overlap among OCA subtypes, molecular diagnostic approaches assume paramount importance in identifying precise gene mutations and discerning OCA subtypes.

Here, we report two unrelated Caucasian patients of Croatian ancestry with different OCA phenotypes and the same genotype; the patients are heterozygous for *TYR* c.1A>G, p. (Met1?), which is pathogenic, and homozygous for *TYR* c.1205G>A, p. (Arg402Gln), which is classified as a risk factor.

## 2. Case Presentation

### 2.1. Case 1

The first patient, a 5-year-old boy with nystagmus, was referred to our Reference Center for clinical examination and genetic testing. At the age of 6 months, parents noticed the “flickering“ of the eyes. His grandfather, great-grandfather, and great-grandmother on the mother’s side were extremely fair-skinned, and his sister is also fair-skinned and wears glasses. His best-corrected visual acuity (BCVA) tested with Lea Symbols inline chart at 3 m measured 0.6 logMAR binocularly, while this tested monocularly 0.7 logMAR on the right eye (RE) and 0.6 logMAR on the left eye (LE). When reading binocularly, a face turn to the left was evident. When tested at 40 cm, the BCVA measured 0.4 logMAR binocularly, while monocularly measured 0.3 logMAR on the RE and 0.4 logMAR on the LE. Retinoscopy demonstrated a refractive error of +0.50 Dsph/ +2.00 Dcyl x 70° on the RE and +0.25 Dsph/+1.50 Dcyl x 110° on the LE. The Lang test was positive and the Titmus fly test demonstrated fine local stereopsis of 100 s of arc. A clinical examination revealed very light pigmented skin and hair. Furthermore, extremely low-amplitude and low-frequency nystagmus was noticeable, with a zero point in convergence, strongly intensifying in levoversion and significantly lessening in dextroversion.

Biomicroscopy revealed marked photophobia, but brown pigmented irises with no transillumination.

Farnsworth’s D-15 dichotomous test and Lanthony desaturated 15-hue panel revealed no dyschromatopsia. The CSV-1000 contrast sensitivity test for the spatial frequencies of 3, 6, 12, and 18 cpd was slightly reduced to 1.63 log units, 1.84 log units, 1.40 log units, and 0.96 log units when tested binocularly and monocularly with the RE, while, when tested with the LE, measured 1.63 log units, 1.84 log units, 1.54 log units, and 0.96 log units.

Goldmann visual field tested with I4e revealed no scotomata with a sum of meridians 1068° on the RE and 1081° on the LE.

**Figure 1:**
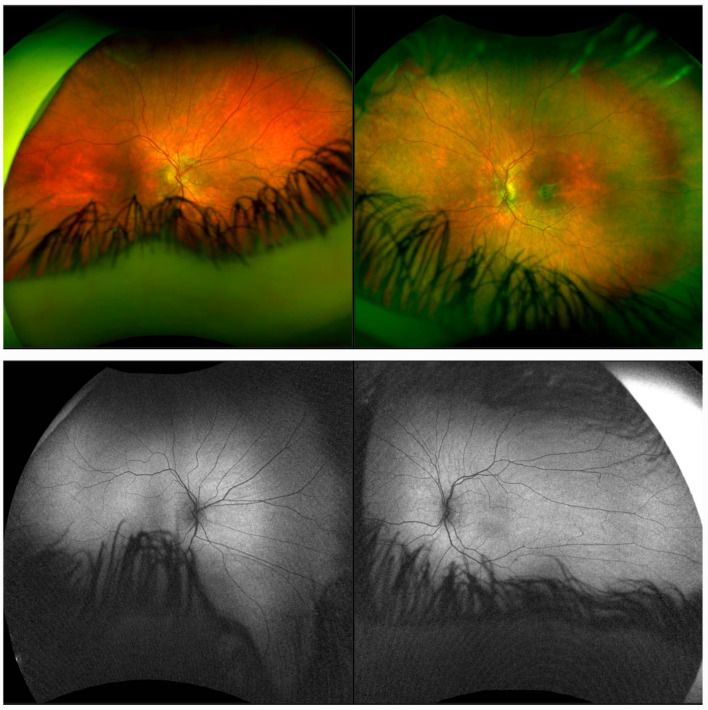
Ultra-widefield imaging depicted brightly pigmented retina on both eyes, consistent with grade 1 fundi (**top row**). Fundus autofluorescence image of the both eyes was isoautofluorescent (**bottom row**).

Optos^®^ California (Optos Inc., Marlborough, MA, USA) ultra-widefield imaging depicted optic nerve heads with clear boundaries and that the entire retina was hypopigmented, while the macular area showed more pronounced pigmentation, but was coarsely granular, as consistent with grade 1 fundi [11] (Figure 1). Fundus autofluorescence (FAF) was isoautoflurescent (Figure 1).

**Figure 2:**
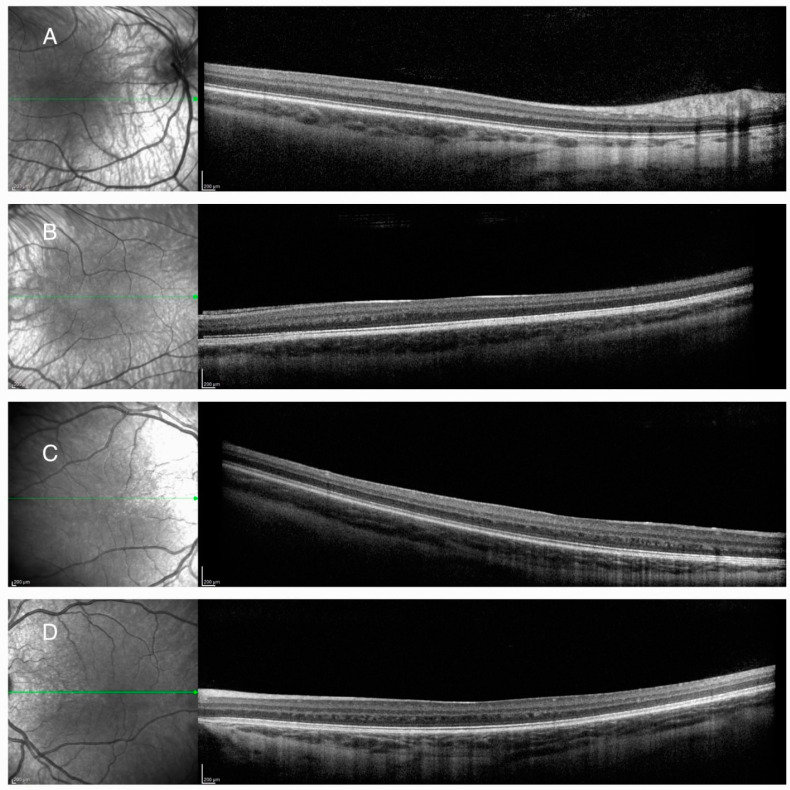
Optical coherence tomography (OCT) imaging showing the right (**A**) and left (**B**) macular area of the first patient and the right (**C**) and left (**D**) macular area of the second patient. Foveal hypoplasia grade 4 according to the Leicester Grading System was evident.

HRA + OCT Spectralis^®^ (Heidelberg Engineering, Heidelberg, Germany) optical coherence tomography (OCT) imaging depicted FH grade 4 according to the Leicester Grading System for FH on both eyes (BE) (Figure 2A,B) [12], with central macular thicknesses of 320 μm and 305 μm on the RE and LE, respectively.

Pattern reversal visual evoked potential (p-VEP) albino protocol testing (Roland Consult RETIport/ scan 21, Roland Consult Stasche and Finger GmbH–German Engineering, Brandenburg an der Havel, Germany), performed using goldcup electrodes, revealed the asymmetry of the response that would correspond to the presentation of crossed fibers as part of ocular albinism. Full-field electroretinography (FFERG) showed no gross rod or cone system dysfunction.

### 2.2. Case 2

The second patient was a 3-year-old boy and was referred to our Reference Center for clinical examination and genetic testing. At the age of 2 months, parents noticed convergent strabismus of BE; therefore, alternating occlusion was recommended. Several members of the family, both on the father’s and on the mother’s side, were extremely fair-skinned. The patient was not related to the first patient.

His BCVA tested with Lea inline optotypes at 3 m binocularly measured 0.4 logMAR, while, when tested monocularly, on the RE measured 0.5 logMAR and 0.4 logMAR on the LE. When tested at 40 cm, BCVA measured 0.2 logMAR binocularly, 0.2 logMAR monocularly on the RE, and 0.3 logMAR on the LE. Retinoscopy demonstrated a refractive error of +2.00 Dsph/+1.00 Dcyl x 100° on the RE and +1.00 Dsph/+0.25 Dcyl x 90° on the LE. Lang and Titmus tests demonstrated no evidence of stereopsis.

Upon clinical examination, marked photophobia with light pigmented skin and hair were evident. Anomalous head posture was present as follows: discrete levoversion with the head tilting to the right shoulder. In the opposite position, nystagmus of low amplitude and medium frequency, oriented in the direction of the fixation (dextroversion), could be elicited. In convergence, the same occurrence of nystagmus was evident, while the cover test was positive for alternating esotropia. Eye motility was otherwise unremarkable.

Biomicroscopy revealed extremely lightly pigmented irises (light blue), but the rest of the anterior segment exam was unremarkable. No iris translucency could be seen in retroillumination.

**Figure 3:**
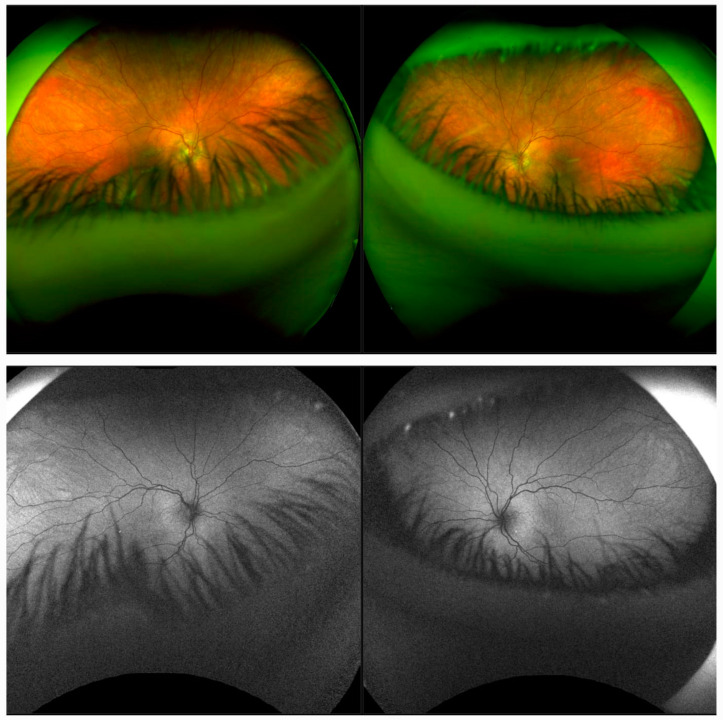
Ultra-widefield imaging showing pronounced light pigmented retina consistent with grade 2 fundi and absent macular reflexes on both eyes (**top row**). Fundus autofluorescence image showing isoautofluorescent signal on both eyes (**bottom row**).

Optos^®^ California ultra-widefield imaging showed pronounced light pigmented retina (grade 2) with absent macular reflexes [11], while FAF revealed hypopigmented RPE with an isoautofluorescent signal (Figure 3).

HRA + OCT Spectralis^®^ OCT imaging depicted foveal hypoplasia grade 4 on BE (Figure 2C,D) [12], with a central macular thickness of 319 μm on the RE and 313 μm on the LE.

The p-VEP albino protocol testing using goldcup electrodes was not feasible due to the patient’s lack of cooperation.

Saliva samples from both patients were collected for genetic testing, and sequence analysis using the Blueprint Genetics Retinal Dystrophy Panel Plus (version 7, 30 October 2021) identified that the patients were heterozygous for *TYR* c.1A>G, p. (Met1?), which is pathogenic, and homozygous for *TYR* c.1205G>A, p. (Arg402Gln), which was classified as a risk factor.

In the context of genetic analysis, the examination of the two patients revealed heterozygous variants of uncertain significance (VUS) in several genes associated with retinal and cellular functions. Patient 1 carries a heterozygous variant in *CFAP410* (c.224C>T, p. (Pro75Leu)), identified as a VUS. Patient 2 presents with several heterozygous VUS variants as follows: *ABCA4* (c.2878G>A, p. (Ala960Thr)), *AGBL5* (c.2360G>T, p. (Arg787Met)), *ALMS1* (c.9463A>T, p. (Thr3155Ser)), *PDE6B* (c.2174C>T, p. (Pro725Leu)), and *INPP5E* (c.875G>A, p. (Arg292His)). None of the genes listed (*CFAP410*, *ABCA4*, *AGBL5*, *ALMS1*, *PDE6B*, *INPP5E*) are known to be directly responsible for eye, skin, or hair pigmentation/color, and are thus unlikely to exert influence on pigmentation traits in eye, skin, or hair coloration in the cases presented.

## 3. Discussion

### 3.1. TYR Gene

The *TYR* gene encodes tyrosinase, an enzyme that participates in the catalysis of the conversion of tyrosine to melanin. Tyrosinase catalyzes the first two steps and at least one subsequent step in this conversion [13]. Pathogenic variants in *TYR* cause autosomal recessive OCA type 1A (OCA1A) and 1B (OCA1B). OCA1A is characterized by a complete lack of tyrosinase activity due to the production of an inactive enzyme, and type OCA1B is characterized by reduced activity of tyrosinase [14]. Frequencies of disease-causing variants in the major causative OCA genes are estimated as follows: *TYR* (44%), *OCA2* (17%), *TYRP1* (1%), *SLC45A2* (7%), and *SLC24A5* (<0.5%) [15]. There are currently over 540 variants in *TYR* annotated as disease-causing in the HGMD Professional variant database (version 2023.3) [16], contributing to a wide genetic heterogeneity. The majority are missense variants, but also include the following truncating variants: nonsense, frameshift, variants affecting splicing, and gross deletions. The vast majority of the variants are reported in association with *TYR*, which is also the most common subtype found in Caucasians and accounts for about 50% of albinism cases worldwide [17,18]. Most individuals with OCA1 are compound heterozygotes with different paternal and maternal pathogenic *TYR* variants [19].

### 3.2. TYR c.1A>G, p. (Met1?)

This variant disrupts the canonical start codon of transcript NM_000372.5, leading to a failure of protein translation or to the formation of an abnormal protein due to the use of an alternative start site. This variant is a loss-of-function variant in the gene *TYR*, which is intolerant [20]. The *TYR* p. (Met1?) variant has been described in patients with autosomal recessive OCA1 [21,22,23,24,25,26]. In gnomAD, there are 19 individuals heterozygous for this variant [27]. The variant is observed in 0.0132% alleles from individuals of European (Non-Finnish) backgrounds in gnomAD [27]. The variant is predicted to be damaging by both SIFT and PolyPhen2 [20]. The following American College of Medical Genetics (ACMG) criteria were applied in classifying this variant as pathogenic: PVS1, PM2, PP3, PP4, and PS1 [28].

### 3.3. TYR c.1205G>A, p. (Arg402Gln)

The *TYR c.1205G>A*, p. (Arg402Gln) variant has been reported several times in the literature, but the disease-causing role of this variant is not clear. In ClinVar, conflicting classifications were identified for this variant as follows: pathogenic (1), uncertain significance (5), likely benign (2), and benign (1) [29].

The variant was found at an allele frequency of 0.2465, predominantly in the European-Non Finnish population [30]. This results in a conservative amino acid change within the tyrosinase copper-binding domain of the encoded protein sequence, outside of the splicing consensus sequence [29]. In silico and computational prediction software programs do not predict a deleterious effect on splicing [30], hence it could be classified as benign [29]. By itself, this variant is not sufficient to cause albinism, since individuals who are homozygous for *TYR* c.1205G>A, p. (Arg402Gln) are unaffected and do not show clinical signs of albinism [29]. However, when in a compound heterozygous state with a loss-of-function tyrosine change, c.1205G>A is acting as a hypomorphic variant, causing a mild form of OCA1B or autosomal recessive ocular albinism (AROA) phenotype [17,18,31,32,33,34,35]. The phenotypic analysis of 69 compound heterozygous patients with the *TYR* c.1205G>A, p. (Arg402Gln) variant, displaying ocular features of albinism, revealed predominant traits including blue irises (76.56%), white or yellow-white hair at birth (71.43%), transitioning to blond hair later (46.97%), and generally light skin with residual pigmentation exceeding that typically observed in classical OCA1 cases (69.64%) [31]. Opposed to primarily manifested milder forms of albinism with atypical clinical presentations, in some cases where this variant was present in trans with a pathogenic variant, clinical symptoms were absent [36]. This observation suggests a potential for the underdiagnosis of affected individuals [30]. Therefore, it has been proposed that the p. Arg402Gln variant causes a partial albinism phenotype, but only when paired with certain genetic backgrounds [32,33].

### 3.4. Genotype-Phenotype Correlation Model

In this study, we present the cases of two male individuals sharing identical genotypes yet exhibiting contrasting phenotypic manifestations in terms of eye, skin, and hair coloration. Specifically, the younger patient, aged 3 years, presented with blond hair and blue eyes (Fitzpatrick skin type II), while the elder 5-year-old counterpart displayed brown eyes and light-brown hair (Fitzpatrick skin type III) [37]. In previous research, *OCA2* was traditionally perceived as the primary genetic determinant of eye color [38]. However, subsequent investigations have elucidated the significant role of the *HERC2* gene, located in close proximity to *OCA2*, in the regulation of iris pigmentation, thereby influencing the expression of *OCA2* [39,40]. Remarkably, the *HERC2-OCA2* locus exerts the most substantial genetic influence on eye color, wherein intronic single nucleotide polymorphisms (SNPs) within *HERC2* interface with the promoter region of *OCA2* through chromatin-loop remodeling mechanisms [41]. It is intriguing to note that variations in DNA associated with eye color predominantly reside within non-coding regions of the implicated genes, namely *TYR*, *TYRP1*, *SLC45A2*, *SLC24A4*, *SLC24A5*, *ASIP*, *MC1R*, and *IRF4*. In this context, the potential impact of tyrosinase activity on these phenotypes warrants consideration [41]. Notably, the polymorphic variant *TYR* c.1205G>A, p. (Arg402Gln) encodes a tyrosinase with thermosensitive properties, displaying only 25% of the normal catalytic activity at 37 °C, owing to its retention within the endoplasmic reticulum [42,43]. Yet, it becomes functional at temperatures between 31 °C and 32 °C [42,44]. As a result, the most severe pigmentary deficiency is expected to occur in tissues located near the core body temperature, such as the eye and optic neural tracts [42,43]. In light of these observations, we hypothesize that environmental variables, including geographic location and climatic conditions, may exert an influential role. The blue-eyed individual originates from a geographic region characterized by a hot Mediterranean climate situated in the far south reaches of Croatia, whereas the brown-eyed subject originates from a northern part of Croatia characterized by a continental, colder climate.

In addition to previous observations posited by other authors, which suggest a direct correlation between BCVA and the degree of FH [45,46], this case study introduces a novel perspective. It is noteworthy that both individuals under our investigation display type 4 FH. Thus, we propose that the variances in pigmentation levels within ocular structures may play a pivotal role in shaping and facilitating optimal visual development. Our findings propose the potential association between reduced pigmentation levels in ocular tissues and binocular functions, suggesting pigmentation as a possible independent variable influencing the onset of strabismus. As pigment within the RPE and choroid exerts a pivotal role in regulating light transmission and absorption within the eye, alterations in pigmentation levels have the potential to modulate the distribution of light reaching the retina, thus reducing light scatter and related glare. This modulation can impact visual perception and potentially influence the development of ocular motor control mechanisms involved in eye alignment. Additionally, melanin content has been implicated in regulating ocular growth and development [47]. Our case study provides empirical support for these assertions. Specifically, the brown-eyed subject presented with astigmatic refractive error and both global and local stereopsis capabilities, contrasting with the presentation of hypermetropia, strabismus, and no stereopsis in the blue-eyed individual. However, the blue-eyed individual, being in a younger developmental stage, has not yet completed the emmetropization phase, implying that further modifications in refractive error are anticipated. Nonetheless, the presence of strabismus indicates a pathological element in visual development that is unrelated to the specific timing within the emmetropization process. Hence, variations in pigmentation levels have the potential to influence the structural development of neural pathways governing eye movement control, thereby predisposing individuals to strabismus.

Within the *TYR* locus, polymorphisms such as *TYR c.1205G>A* variant in exon 4, translating to Arg402Gln, are prevalent, yet display complex relationships with pigmentation phenotypes [28,29,30,31,32,33,34,35,36]. Herein, we propose a genotype–phenotype correlation model to elucidate the diverse clinical presentations stemming from biallelic and triallelic pathogenic variants in *TYR*, establishing a link between residual tyrosinase activity and resultant phenotypes.

Similar genetic complexity, albeit unrelated to albinism, is observed in Stargardt Disease Type 1 (STGD1), a juvenile macular dystrophy caused by biallelic variants in *ABCA4* [48,49]. This disorder manifests a spectrum of inherited retinal dystrophy phenotypes, encompassing conditions such as cone-rod dystrophy (CRD), atypical retinitis pigmentosa (RP), fundus flavimaculatus, generalized choriocapillaris dystrophy, and rapid-onset chorioretinopathy (ROC) [48,49,50,51]. The specific clinical presentations depend on the combination and severity of the genetic variants involved; severe null alleles in a biallelic configuration typically present as atypical RP or ROC, while severe alleles in trans with moderate variants tend to result in CRD. Combinations of severe with mild or two moderate variants often lead to the manifestation of STGD1 [50,51].

According to our proposed model, the severity of *TYR* variants correlates with distinct albino phenotypes. Due to the reduced total tyrosinase catalytic activity, homozygosity for the p. (Arg402Gln) allele does not result in disease manifestation [29]. The compound heterozygosity for severe *TYR* mutations and the common polymorphic p. (Arg402Gln) variant, occurring at an approximate frequency of 1 per 280 Caucasian individuals [34], manifests as AROA. However, when homozygosity for the p. (Arg402Gln) allele is combined with a functionally significant variant in a triallelic configuration, all individuals exhibit the true OCA phenotype, with the severity being modulated by the variant’s impact and the degree of tyrosinase activity reduction in the heterozygous state.

## 4. Conclusions

Our patients display compound heterozygosity for the complex allele p. [Met1?;Arg402Gln] and the hypomorphic variant p. (Arg402Gln), characterized by heterozygosity for *TYR* c.1A>G, p. (Met1?) and homozygosity for *TYR* c.1205G>A, p. (Arg402Gln). Despite possessing identical genotypes, they exhibit divergent phenotypic expressions, a phenomenon that we hypothesize is potentially influenced by distinct climatic environments. Furthermore, our observations propose a plausible association between reduced pigmentation levels in ocular tissues and binocular functions, positing pigmentation as an independent variable influencing the onset of strabismus—an association until now unreported in the existing literature, thereby warranting further investigation.

*TYR* c.1A>G, p. (Met1?) is classified as pathogenic based on the currently available evidence supporting its disease-causing role. On the other hand, the disease-causing role of *TYR* c.1205G>A, p. (Arg402Gln) remains ambiguous, but it has been reported to be enriched in albinism patients with only one disease causing *TYR* variant identified.

We propose a model suggesting that the severity of the polymorphic *TYR* c.1205G>A, p. (Arg402Gln) variant is linked to specific albino phenotypes, depending on the pathogenicity of the trans variant as follows:

- Homozygosity for the p. (Arg402Gln) allele does not manifest the disease [29] due to adequate persistent tyrosinase activity;
- Compound heterozygosity involving severe *TYR* mutations and the common p. (Arg402Gln) polymorphism, occurring at a frequency of approximately 1 in 280 Caucasians [34], results in AROA;
- When homozygosity for the p. (Arg402Gln) allele is combined with a functionally significant variant in a triallelic configuration, the classical OCA phenotype is invariably expressed.

## Data Availability

The data presented in this study are available on request from the corresponding author. The data are not publicly available due to privacy protection.
